# An Account of the Good Effects of Copious Blood-Letting in the Cure of an Hemorrhage from the Lungs; in a Letter from the Rev. Dr. Samuel Smith, President of the College of New Jersey, to Dr. Benjamin Rush

**Published:** 1807-10

**Authors:** 


### *An Account of the good Effects of copious Blood-letting in the Cure of an Hemorrhage from the Lungs; in a Letter from the* Rev. Dr. Samuel Smith, *President of the College of New Jersey, to* Dr. Benjamin Rush.

Dear Sir,

As the science of medicine is established on an induction of facts, well attested histories of the successful treatment of diseases must be singularly useful. It is with a view to add one case more to your useful collection, that I have been induced to give you an account of an uncommon hemorrhage with which I have been affected myself, and of my perfect recovery from it.

A certain weakness of breast, and tendency to hemorrhage, was hereditary on my mother's side of the family, whom I was supposed to resemble in constitution and countenance. Shortly after I was licensed to preach I was thrown into a situation which required unusual exertions. I had often to address very large assemblies, at least three times in the week, and frequently every day. In consequence of these efforts, at the end of about three years I began to raise small quantities of blood at the close of each discourse. This symptom continued to increase; and during nearly four years more, though I moderated my exertions, and made them much less frequent,

I raised

I raised some blood at the end of almost every sermon that I delivered, and sometimes in considerable quantities. The spitting often continued, in a less or greater degree, for several days afterwards. I was at length obliged to intermit preaching entirely for the space of eight months, during which time I spent part of a season at the Sweet Springs in Virginia. I recovered a tolerable, though a delicate state of health. At this period I was removed to Princeton, where speaking to small assemblies, and in a chapel happily accommodated to favour the voice, I continued to preach, and to attend my other duties in the College, during two years, with increasing health and vigour. In the autumn of the year 1782, I suddenly broke a blood-vessel in my breast, as I was walking. A considerable quantity of blood issued from the wound; but by bleeding copiously at my arm, the flux from my breast at that time was stopped. Nearly about the same time in the next evening I felt my pulse quicken, and an unusual tension growing on all my nerves, and, in a few seconds, the blood began to spout with great velocity thro' my mouth and nose; I was again bled largely twice in the course of the evening, from my arm and foot. The following evening a similar paroxysm returned, and the blood again spouted with great force from my mouth. The physician was sitting on my bed-side at the time; and when I perceived the quickening of my pulse, and that strange stricture coming, as it seemed to my feeling, on the whole system of the nerves, I gave him notice of it, and requested him to bleed me. He refused, and said, so much bleeding would only tend to bring on an habitual hemorrhage by debility. I told him, I would rather die bleeding from the arm than the mouth. At last, when he saw the force with which the blood issued, and, in consequence of my earnest solicitations, he bled me again. I requested him to leave me his lancet, and I would be answerable for my own life. This he reluctantly consented to do. At this time two other physicians were called in, who bled me twice more the same evening, before they could effectually stop the flux from my mouth. Next day I expectorated nearly a pint of clotted blood, that had been lodged somewhere in the cavities of the lungs. Being now in possession of a lancet, and apprised by experience of the symptoms that preceded the return of my disorder, I determined to anticipate it, and when the physicians were not present, to open my own veins. This I did accordingly, whenever I perceived the symptoms I have already mentioned, mentioned, which now recurred more frequently for four or five days. After that period they subsided. But I continued bleeding at the arm two, three, and four times in a day, until the tenth day. My intention was to take oft the impulse of the blood from the wounded part, to prevent an inflammation, and to give it time to heal. I may, perhaps, have bled myself oftener than was necessary.— After pursuing this course, however, the flux of blood no more discovered itself from my breast. Two-and-thirty times I was bled in the space of ten days, in my arms and feet, besides what flowed from my mouth. And it was computed, from the dimensions of the vessels in which it was received, and other circumstances, that I lost, in that time, at least two gallons of blood. Even in this reduced state, so great was my apprehension of the hemorrhage in my breast returning, I continued to bleed at the arm twice in the week for some time, afterwards once in the week, and finally, once in a month during several months; tho' not in large quantities at each time. After the hemorrhage was entirely stopped my flesh wasted away for want of its proper nourishment in the blood, till not a muscle could be perceived on any of my limbs; the skin appeared to be drawn almost close to the skeleton; I spoke only in a whisper; and I was not sensible of having forgotten myself for a moment in sleep during six weeks. My complexion was tinged with a yellowish hue, and an acid so prevailed in my constitution, that with my tongue I have frequently curdled a small bowl of milk. For more than three months I could not use bread, or any vegetable substance in my diet. I lived chiefly on beef liquor, soups, and white meats. I drank a little weak wine and water frequently; but after many trials, I found porter as a drink agree best with my stomach, and at the end of some months I used it freely and constantly. As soon as I could bear the motion of a carriage, I took gentle exercise in that way. Afterwards, when I could sit a horse alone, I was helped into the saddle, and every day rode a small distance, increasing it gradually till I rode from twelve to fifteen miles in a day. Here my recovery was at a stand for some months, when I resolved to try the effect of a long journey on horseback. I rode first into Connecticut, and afterwards to Boston, in which journeys I laid the foundation of that good health which I have now continued to enjoy for more than ten years; and my voice, particularly, is clearer and stronger than it ever was. A habit that frequently required aperient medicines, was greatly increased by by loss of blood, and for more than seven years I was obliged to have daily recourse to them. It is now upwards of three years since I have used them at all. A pint of beer or of cyder at any time sufficiently answers the purpose. This fact is contrary to another principle of many physicians, and contrary to the pressing advice of several of my medical friends, rather to struggle with the disease than to use so constantly the medicines necessary to overcome it. They assured me that I would totally destroy the tone of my bowels, and perhaps fall an early victim to my remedies. However, resolved to live as comfortably as possible while I should live, and not solicitous for a prolonged existence of sickness and pain, I persisted in my own course; and I now find that the medicine was infinitely less pernicious than the habit, and that, contrary to prediction, it has relaxed the tone of my bowels just to that state in which it ought to be. I have found, however, many years ago, that a small pressure upon them, impeded their regular action, and produced sickness at stomach, and pain in the head. This induced me to invent a kind of suspenders to relieve my stomach, long before I knew that fashion would employ them to shew the shape to more advantage. My own experience has convinced me, that persons of delicate habits often suffer greatly from tight waist-bands, when they are not able to assign the cause, and especially women, from the manner in which many of them tie their clothes, and the weights they suspend in their pockets.

Thus, Sir, I have given you the history of my disease, and the treatment of it, which, I doubt not, you will pronounce uncommon. I have gone back to an early period of it, that, having all the facts before you, you might form a more accurate judgment of the whole. I have, perhaps, carried my bleedings somewhat farther than was absolutely necessary; but, in such cases, it is difficult to fix the point of strict necessity, and success has justified my rashness. And I am persuaded that, to great bleeding, and the thick flannels in which I immediately wrapped myself, all with a view of taking off the impulse of the blood from the wound in my breast, I owe my freedom almost wholly from inflammation and cough. I judge thus, because in many instances since that time, when I have imprudently taken cold, and feared the effect of the cough on my breast, I have had immediate recourse to increase of flannel and to bleeding, which have relieved me in a very short time.

I have

I have had no medical education, and have had no theory to bias my mind. What I have done at any time contrary to advice, I have done, impelled by the urgency of symptoms, or led by probable conjectures concerning the effect of the means I used, without being restrained by Principles which might, perhaps, have intimidated me. I draw no general conclusions from my particular case. This you will be better able to do when you have compared it with so many others which must have come under your observation. I believe it would be dangerous for an indolent and inactive person to be reduced so low by bleeding as I have been, or one who had not equal resolution to make the exertions necessary to recover himself from that state. Activity in duty and firmness of mind, are often among the best medicines.

